# Dynamics of fungal endophytic communities in bilberry (*Vaccinium myrtillus* L.) fruits through development is shaped by host phenolic compounds

**DOI:** 10.1093/femsec/fiae168

**Published:** 2024-12-23

**Authors:** Minh-Phuong Nguyen, Kaisa Lehosmaa, Françoise Martz, Janne J Koskimäki, Katalin Toth, Saija H K Ahonen, Hely Häggman, Anna-Maria Pirttilä

**Affiliations:** Ecology and Genetics Research Unit, PO Box 3000, University of Oulu, FI-90014 Oulu, Finland; Ecology and Genetics Research Unit, PO Box 3000, University of Oulu, FI-90014 Oulu, Finland; Natural Resources Institute Finland, Production Systems, Ounasjoentie 6, FI-96200 Rovaniemi, Finland; Ecology and Genetics Research Unit, PO Box 3000, University of Oulu, FI-90014 Oulu, Finland; Inari Agriculture Nv, Industriepark Zwijnaarde 7a, 9052 Gent, Belgium; Ecology and Genetics Research Unit, PO Box 3000, University of Oulu, FI-90014 Oulu, Finland; Ecology and Genetics Research Unit, PO Box 3000, University of Oulu, FI-90014 Oulu, Finland; Ecology and Genetics Research Unit, PO Box 3000, University of Oulu, FI-90014 Oulu, Finland

**Keywords:** food microbiology, fungal endophyte, microbial ecology, host-specific, metabolic crosstalk, phenolic compounds, plant development

## Abstract

The physical and chemical properties of wild berry fruits change dramatically during development, and the ripe berries host species-specific endophytic communities. However, the development of fungal endophytic communities during berry ripening is unknown. We studied bilberries (*Vaccinium myrtillus* L.), valuable natural resources in northern Europe and richest sources of phenolic compounds, to characterize dynamics of the fungal communities over fruit developmental stages (raw, veraison, and ripe). Our focus was to examine the changes in the fruit phenolic compounds associated with the fungal community structure using liquid chromatography–mass spectrometry for phenolic compounds and high-throughput sequencing technology targeting the internal transcribed spacer 2 ribosomal DNA region for endophytic fungi. We found that the fungal diversity increased with the ripening stages. The fungal profile changed dramatically through fruit development, and the veraison stage was a transition stage, where the core mycobiome of fruits changed. The fungal community structure and abundance of the most dominant genera in raw and ripe stages, *Monilinia* and *Cladosporium*, respectively, were driven by the bilberry phenolic profile. We conclude that sampling time, tissue age, and phenolic compounds play important roles in the development of fruit fungal community. Moreover, phenolic compounds could be the host's strategy to recruit beneficial microbes.

## Teaser

The diversity and profile of fungal endophytic communities change dramatically through bilberry fruit development; sampling time, tissue age, and host phenolic compounds shape the fungal communities.

## Introduction

Plants can be considered holobionts (a macrobe and its microbial associates), in which the overall stability of the system depends on all interacting organisms (Vandenkoornhuyse et al. 2015). Healthy plants harbor diverse communities of microorganisms both inside their tissues (endophytes) and on their surface (epiphytes). The gene pool of these microorganisms extends the host genome and contributes to the phenotype, affecting the fitness and adaptation of the host (Müller et al. 2016). Compared to epiphytes, endophytes can benefit the host in various ways (Hardoim et al. 2015) through the close relationship. For example, fungal endophytes can enhance the plant tolerance against several abiotic stresses, such as drought (Baltruschat et al. 2008), salinity (Waller et al. 2005), and nutrient deficiency (Rho et al. 2018), as well as biotic stress, such as plant pathogens and herbivores (Arnold et al. 2003, Gunatilaka 2006, Tejesvi et al. 2013). Endophytes can modify the secondary metabolism of inoculated plants (Koskimäki et al. 2009, Lòpez-Fernàndez et al. 2016, Yang et al. 2016, Pan et al. 2020), or improve the fruit biomass (Fakhro et al. 2010). Factors affecting the lifestyle of endophytes include the host genotype and fitness (Hardoim et al. 2015), their interaction with the host plant (Collinge et al. 2022), and the structure of the full endophytic community (Martins et al. 2021). Therefore, a balanced microbiome is required for optimal plant health. An understanding of the formation of endophytic communities and their underlying factors is needed to enhance applications of endophytes in crop management strategies against plant diseases, to improve fruit quality, and to control postharvest diseases (Lugtenberg et al. 2016).

So far, endophytic microbiome studies in wild berry species have mostly concentrated on the vegetative parts and specific endophytes (Fisher et al. 1984, Xu et al. 2007, Koskimäki et al. 2009, Tejesvi et al. 2016, Yurgel et al. 2018). Studies focusing on fruit microbiome are rarely done, and mostly concentrate on commercial species, such as cranberry (Tadych et al. 2012), apple, and pear (Glushakova and Kachalkin 2017). Our earlier study on three wild berry species showed that the majority of cultivable fruit microbiome is comprised of fungi, and that the fruit-associated endophytes are mainly species-specific and horizontally transmitted, similar to foliar endophytes (Nguyen et al. 2021). However, the knowledge on the impact of fruit secondary metabolites on the development of endophytic communities is lacking, although the fruit metabolic profile changes dramatically throughout the ripening process (Jaakola et al. 2002, Suvanto et al. 2020). Fruits are typically rich in secondary metabolites, especially phenolic compounds, such as phenolic acids, hydroxycinnamic acids, flavonoids, proanthocyanidins, lignans, and tannins (Lattanzio 2013). Many phenolics are highly antioxidative and possess antimicrobial activities. Therefore, they are important factors in the plant defense against abiotic and biotic stresses (Dixon 2001), including colonization by endophytes. For example, the plant family Leguminosae and Vitaceae produce isoflavonoids and stilbene as phytoalexins against fungal parasites (Lattanzio 2013). In general, flavan-3-ols (e.g. catechin and epicatechin) and tannins are suggested in plant defense against fungal pathogens (Goetz et al. 1999, Hébert et al. 2002, Koskimäki et al. 2009, Wang et al. 2017). Plant chemical traits affecting endophytic colonization have earlier been studied mainly in leaves (Schulz and Boyle 2005, Saunders and Kohn 2009). Exploring the interaction between fruit phenolics and fruit-associated endophytic fungi can shed light on how the host affects the development of symbiotic microbial communities through secondary metabolism.

Bilberry (*Vaccinium myrtillus* L.) is an important wild berry species in northern Europe due to its abundance in nature, health-beneficial properties as a functional food, and importance in economy (Chu et al. 2011, Zoratti et al. 2015). Picking of bilberries is a valuable economic source for many regions in the Nordic countries due to the right of public access to all forests (Turtiainen and Nuutinen 2012). Between 2016 and 2020, an average value of exported bilberries was 6.4 million euros (equal to 2.3 million kg of fresh and frozen bilberries) (Miina et al. 2021). Bilberry fruits are consumed fresh, frozen, dried, or used as food products (e.g. juices and jams) and dietary supplements (Salo et al. 2021), and are also a potential source for extraction of bioactive compounds, such as anthocyanins and waxes (Trivedi et al. 2019). Bilberries contain an exceptionally high content of flavonoids, especially anthocyanins both in the skin and the flesh (Jaakola et al. 2002, Heinonen 2007, Lätti et al. 2008, Dare et al. 2022), phenolic acids, and stilbenes (Može et al. 2011). Anthocyanins are powerful antioxidants with activity against serious health disorders, such as neuronal and cardiovascular disorders, cancer, and diabetes (Zafra-Stone et al. 2007). The quality of phenolics make bilberry a valuable therapeutic food (Zafra-Stone et al. 2007, Chu et al. 2011). Specifically, bilberry extract and phenolics have shown antimicrobial effects against human pathogens (Puupponen-Pimiä et al. 2005a,b). Several studies have shown that the composition of flavonoids in bilberry fruit is strongly affected by the fruit developmental stage (Jaakola et al. 2002, 2010) along with genetic and environmental factors (Lätti et al. 2008, Åkerström et al. 2010, Uleberg et al. 2012, Zoratti et al. 2015).

The high richness of phenolic compounds and the drastic change of the phenolic profile during bilberry fruit development raise the question on how endophytes colonize such aromatically enriched environment. Phenolic compounds not only play important roles in signaling and plant defense against microbes (Mandal et al. 2010, Lattanzio 2013), but they are also involved in the interaction with endophytes via metabolic crosstalk (Hardoim et al. 2015, Ludwig-Müller 2015). Therefore, studying the endophytic community structure through fruit ripening stages will provide valuable insights into the development of the endophytic communities in response to accumulating phenolic compounds. In this study, we selected three ripening stages of bilberry fruits to follow the development of fungal microbiome: when the fruits were growing (raw, green berries), changing color (veraison, red berries), and fully ripening (ripe, blue berries). We aimed to answer the following questions: (i) How does the diversity of bilberry fungal microbiome change over the ripening stages? (ii) Is the core mycobiome persistent throughout the stages? (iii) Do fruit phenolic compounds shape the fungal communities? Based on our earlier results (Nguyen et al. 2021), we expected the fruit mycobiome to follow similar patterns as those found for developing leaves, and the phenolic profile of bilberry to influence the endophytic community composition.

## Materials and methods

### Sample collection

We collected bilberry fruits from two locations in the subarctic Oulu region, northern Finland during the summer of 2021. The locations were selected based on the distance to urban areas and the same forest type. Both selected sampling locations had similar soil properties (Fig. S1 and Table S1). The distance between the locations was ∼3.5 km. Location A (65°05′12.4″N 25°29′39.3″E) is >1 km away from suburban area, and location B (65°05′46.5″N 25°34′14.9″E) is ≥200 m away from the road and 1.5 km away from the suburban areas (see Fig. S2).

We collected berries from three developmental stages: raw, veraison, and ripe. Raw berries were collected on 5 July 2021, while the heterogeneity of the ripening process allowed finding veraison and ripe berries on the same date on 20 July 2021. The sampling sites being public, veraison and ripe berries were collected at the same time to prevent the lack of ripe berries later in the season due to possible berry picking by local people. On each sampling date, six replicates were collected at site A, while only four were collected at site B, due to the lower abundance of berries in the latter location. Each replicate was collected in an area of 4–8 m^2^. A minimum distance of 5 m separated each replicated sampling area. The fruits (ca. 35 berries) were picked using sterile forceps, kept in sterile 50-ml conical tubes, and preserved on ice (+4°C) for transportation and further processing.

### Sample preparation

The fruits were surface sterilized to remove epiphytes and contaminants from the berry skin following the protocol of Nguyen et al. (2021) within 24 h after collection. The surface sterilized berries were stored at −80°C in sterile 50-ml conical tubes until freeze-drying. Approximately 35 berries were pooled together for each sample (10 pooled samples per developmental stage), freeze-dried using a vacuum freeze-dryer, and ground to a fine powder using mortar and pestle under cold and aseptic conditions. From each pooled sample, 65 mg was used for the DNA extraction (one extraction), and 45 mg was used for phenolic extraction (15 mg × 3 replicated extractions).

### Molecular analyses and bioinformatics

DNA was extracted from 65 mg of berry powder with QIAamp Fast DNA Stool Mini kit (Qiagen) using the protocol of Toth et al. (2024). The concentration of extracted DNA was normalized to 40 ng µl^−1^, and 20 µl of DNA was treated with 5 µg of RNase A (10 mg ml^−1^, Thermo Fisher Scientific) for 1 h at 37°C.

Amplification of the fungal internal transcribed spacer 2 (ITS2) of the ribosomal RNA gene region was done in triplicates for each sample in 20-µl reaction containing 200 ng of RNase-treated DNA template, 1X Phusion GC Buffer, 0.2 µM dNTP, 5% DMSO, 10 µg BSA (New England BioLabs), 0.5 µM of each fungi-specific primer fITS7 (5′-**GTGARTCATCGAATCTTTG**-3′) (Ihrmark et al. 2012) and ITS4R (5′-**TCCTCCGCTTATTGATATGC**-3′) (White et al. 1990), and 0.4 U of Phusion High-Fidelity DNA polymerase (Thermo Fisher Scientific). The fITS7 primer had the Ion Torrent sequencing adapter trP1 at the 5′ end, while the ITS4R primers contained 30 bp adapter A with a 10 bp unique barcode for each sample. The polymerase chain reaction (PCR) program included an initial denaturation of 98°C for 3 min, followed by 26 cycles of 98°C for 10 s, 55°C for 20 s, and 72°C for 30 s, and the final extension of 72°C for 7 min. The PCR products were pooled, and their relative quantity was assessed by 1% agarose gel electrophoresis. The samples yielding a very strong band were reamplified with 23 cycles, whereas samples with no visible band were reamplified with 30 cycles. Two identical replicates of a custom-made fungal mock community and negative controls from the DNA extraction and PCR amplification steps were included. The amplicons were purified by magnetic beads with AMPure XP Kit (Beckman Coulter) and quantified with PicoGreen (Invitrogen) according to the manufacturers’ instructions. The cleaned DNA amplicons were equimolarly pooled and sequenced with Ion 316^TM^ chip and Ion Torrent PGM (Thermo Fisher Scientific) technology at the Biocenter Oulu Sequencing Center (Oulu, Finland).

The raw data (2 421 645 single-end reads) were processed with QIIME2 pipeline version 2021.11 (Bolyen et al. 2019) following the procedure suggested by Nguyen (2020) for fungal ITS amplicon data with some modifications. First, the sequences were demultiplexed and primer trimmed using CUTADAPT (Martin 2011), then they were denoised using DADA2 (Callahan et al. 2016) and clustered into operational taxonomic units (OTUs) using VSEARCH at a similarity threshold of 97% (Rognes et al. 2016). The fungal ITS2 regions were extracted using the ITSx software 1.1.3 (Bengtsson-Palme et al. 2013) and then taxonomically assigned in QIIME2 using the UNITE QIIME release for Fungi 2 dynamic database version 8.3 (Abarenkov et al. 2021). Only OTUs having ≥85% match length were retained.

### Phenolic compound analysis liquid chromatography–mass spectrometry

Soluble phenolics were analyzed as previously described by Nguyen et al. (2021). Briefly, soluble phenolics were extracted three times in [methanol:H_2_O (1:1), 0.1% HCl] and analyzed immediately after extraction by using ultra-high-performance liquid chromatography-diode array detection-electrospray ionization-mass spectrometry (UPLC-DAD-ESI-MS/MS, Nexera2, LCMS-8040, Shimadzu, Kyoto, Japan) using a Luna 5 µm C18(2) 100 Å, 250 × 3 mm column (Phenomenex, Torrance, USA) with solvent A (10% methanol and 0.1% formic acid) and solvent B (98% methanol, and 0.1% formic acid) and the following gradient: 0–2 min of 5% B, 15 min of 13% B, 30 min of 40% B, 40–50 min of 100% B (flow 0.3 ml min^−1^, column oven 40°C). The MS conditions were as follows: nebulizing gas (N_2_) 3 l min^−1^, drying gas (N_2_) 15 l min^−1^, desolvation line 250°C, heat block temperature 400°C, and interface voltage 4.5 kV. Quantification was done using MS detection with single ion monitoring and multiple reaction monitoring (MRM) [phenolic acids, flavonoids, proanthocyanidins (PAs), and anthocyanins (ACNs)] in negative or positive modes (Table S2). Quantification by ultraviolet radiation (UV quantification) was used for trans-cinnamic acid (internal standard, 280 nm) and iridoids (IRIs). The quantification of IRIs remained challenging due to the absence of suitable commercial standards. In our study, two major acylated IRIs were identified based on their *m*/*z* and UV spectra (Heffels et al. 2017), with one major compound corresponding to 82% of all IRIs (average over all samples): RT 32.9 min, λ_max_ = 312 nm, [M−H] = 535, [M+formate-H] = 581. All IRIs were quantified with UV detection, using *p*-coumaric acid as a standard, as previously reported (Mikulic‐Petkovsek et al. 2015).

### Statistical analyses

R statistical software environment version 4.2.2 (R Core Team 2021) was used for the analyses. The microbiome data were pre-processed using the *phyloseq* package (McMurdie and Holmes 2013). We subtracted the reads of the negative controls from the samples, removed OTUs having less than six reads, and kept only samples with at least 20 reads. The final processed dataset contained 494 896 reads of 107 taxa and 30 samples (10 raw, 10 veraison, and 10 ripe) with ≥130 reads per sample.

Diversity of the fungal microbiome was analyzed mainly using the *vegan* package (Oksanen et al. 2022). Fungal richness was calculated using the *specnumber* function. To determine whether species richness varied across the developmental stages, we used the Kruskal–Wallis rank sum test, and for pairwise testing we used the pairwise Wilcoxon test with Benjamini–Hochberg correction. Dissimilarity matrix of the fungal OTU table was calculated with a robust Aitchison dissimilarity metric (dRAI) (Tedersoo et al. 2022) using the *vegdist* function. A principal coordinate analysis (PCoA) was conducted on the dRAI matrix using the *wcmdscale* function to visualize the distribution patterns of the bilberry-associated mycobiome. To analyse the variation in community structure (beta diversity) (Anderson 2006), we calculated the homogeneity of multivariate dispersions using the *betadisper* function. Differences in the multivariate dispersion between groups were globally tested with the *permutest* function (9999 permutations), followed by Tukey's pairwise comparisons via the *TukeyHSD* function. Among group differences in the fungal endophyte composition were tested by a permutational analysis of variance (PERMANOVA, 9999 permutations) (Anderson 2001) using the *adonis2* function, specifying “strata=location” to account for replicates at each location. Pairwise PERMANOVAs between stages were run after the global test. Similar methods were applied for the bilberry phenolic profiles to examine their composition grouping by stage. However, a Euclidean distance metric was used on the standardized phenolic data. To summarize the phenolic data, the compounds were grouped into major categories based on their chemical structures. The outlier B-R2 (a veraison sample) was removed from further analyses due to the diversity difference comparison to the average, and A-B3 (a ripe sample) was removed from the phenolic-related analyses.

A relative abundance profile of the fungal communities was examined using the *microeco* package (Liu et al. 2021). Shared and unique OTUs for each developmental stage were visualized using a Venn diagram. The core fungal microbiome was selected based on the 10 most abundant OTUs and 50% prevalence in all samples, or 80% prevalence in each developmental stage (Shade and Stopnisek 2019, Liu and Howell 2021) using the *core* function of the *microbiome* package (Lahti and Shetty 2019). The relative abundance difference among the developmental stages was determined by Kruskal–Wallis with the false discovery rate (FDR)-corrected *P* value of <.05 (Liu and Howell 2021). Dynamics of the core microbiome were visualized by using alluvial diagrams of the *microeco* package.

We tested if the bilberry phenolic profile explained beta diversity and community structure of the fungal endophytes by first applying the principal component analysis (PCA) for the phenolic data to reduce the collinearity between the phenolic variables, and then extracting the eigenvalues of the first PC axis (the phenolic PC1, which explained 77.8% of the variation) for further analysis. The impact of the bilberry phenolic PC1 on the multivariate dispersion of the fungal community (i.e. beta diversity) was examined with a generalized linear mixed model (GLMM) using the *glmmTMB* function of the *glmmTmB* package (Brooks et al. 2017), and the model validation was done using the *DHARMa* package (Hartig 2022). Further, the impact of the change in the phenolic compound profile from the raw to ripe stages (the phenolic PC1) on the structure of the fungal community was determined by applying distance-based redundancy analysis (dbRDA) for the fungal dRAI matrix as the response variable, and the phenolic PC1 as the explanatory variable, using the *dbrda* function of the *vegan* package. In the analysis, our study design was considered by specifying “Condition(location)”. The significance of the dbRDA model and the marginal effect of the terms were tested using permutation tests (9999 permutations), taking into account our study design. In general, dbRDA is a constrained analysis that can reveal whether a matrix of explanatory variables has a significant impact on the (dis)similarities derived from the response data as a whole (Legendre and Anderson 1999) and tells how much of the variation in community structure is explained by functions of other variables (Anderson et al. 2011).

We used the correlation method to show which specific compounds correlate with which fungal genera and generalized-linear mixed models to test if the change in the phenolic compound profile from the raw to ripe stages (the phenolic PC1) affects the abundance of these genera. Based on these methods, it is possible to draw a conclusion if there is a link between the compounds and the fungal genera. Specifically, the correlation between the fungal genera and the standardized berry phenolic compounds was accessed with the Spearman correlation and FDR corrected *P* value of <.05 using the *microeco* package, and significant correlations were depicted with a heatmap. For genera that correlated significantly with the bilberry phenolic profile, GLMM was performed to confirm if the phenolic PC1 drove the abundance of the genera, and the results were visualized by fitting the vectors of the fungal genera on the PCA of the major phenolic groups by using the *envfit* function.

## Results

### Fungal community profiles changed dynamically through fruit development

At all taxonomic levels, the number of taxa was the lowest in the raw stage and the highest in the ripe stage. The phylum Ascomycota was dominant in all developmental stages. The phylum Basidiomycota appeared only in the stages veraison and ripe (veraison: 7%, ripe: <2%). The class Leotiomycetes was the most dominant in the raw and veraison samples and gradually decreased towards the ripe stage (raw: 96%, veraison: 64%, ripe: 37%), whereas Dothideomycetes increased from raw to ripe stages (raw: 3%, veraison: 28%, ripe: 59%) and was the most abundant class in the ripe samples. The number of other classes was the highest in the ripe stage (two in raw, five in veraison, and eight in the ripe stage).

**Figure 1.**
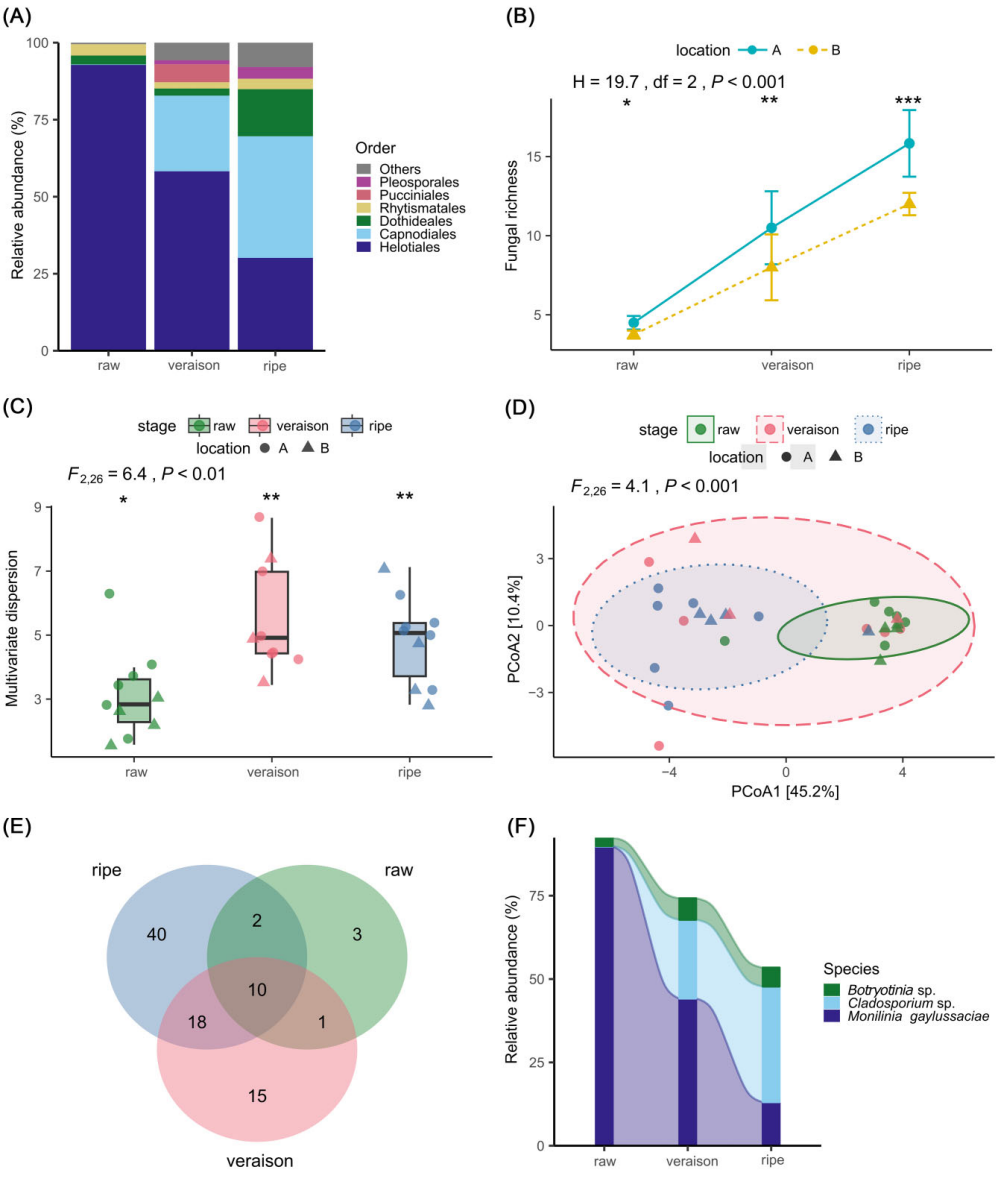
Profile and diversity of the fungal community in different bilberry developmental stages (raw, veraison, ripe). (A) Relative abundance of most dominant fungal orders demonstrated by stacked bar plots, (B) mean and standard error of fungal richness, (C) multivariate dispersion, (D) PCoA ordination visualizing the fungal community assemblages, (E) Venn diagram of the shared and unique OTUs, and (F) alluvial diagram of the core OTUs. Asterisks indicate statistical significance in a pairwise test comparison between stages (in B and C). Ellipses denote a confidence interval of 75% (in D).

At the order level, Helotiales (including the family Sclerotiniaceae and the genus *Monilinia*) was the most abundant in the raw stage (93%) and decreased towards the ripe stage (veraison: 58%, ripe: 30%) (Fig. 1A). Abundance of the Capnodiales (including the family Cladosporiaceae and the genus *Cladosporium*) increased through the developmental stages and reached the maximum at the ripe stage (raw: 0.2%, veraison: 24%, ripe: 39%). Dothideales was also more abundant in the ripe samples compared to the raw and veraison ones (ripe: 15%, raw and veraison: < 3%). The ripe samples also contained the highest abundance of Pleosporales among the three stages, followed by the veraison samples, whereas the raw samples had extremely low abundance of the order (ripe: 3.8%, veraison: 1.3%, raw: <0.01%). The families Dothioraceae (including the genus *Hormonema*), Hyaloscyphaceae (including the genus *Lachum*), and Dothideaceae were more abundant in the ripe developmental samples compared to the raw and veraison ones.

### The fungal diversity and community structure differed among the fruit developmental stages

The fungal richness differed among the three developmental stages and increased along with the fruit development consistently at both sampling locations (Fig. 1B). The pairwise tests for the fungal richness were significant in all comparisons (Wilcoxon, *P* < .05). The multivariate dispersion and community composition also differed among the stages (Fig. 1C and D). However, the pairwise tests for the multivariate dispersion and the community composition were significant between the raw stage and the other stages (*P* < .05) but not between the veraison and ripe stages (*P* > .05). The community composition of raw and ripe stages exhibited the strongest difference (PERMANOVA, *F*_1,18_ = 9.2, *P* < .001), followed by raw and veraison stages (PERMANOVA, *F*_1,17_ = 3, *P* < .05).

The ripe developmental stage had the highest amount of unique OTUs (40 OTUs classified to 23 known genera), followed by the veraison (15 OTUs classified to 11 known genera), and the raw stage (three OTUs classified to two known genera) (Fig. 1E). Ten OTUs were shared among the three developmental stages, and they belonged to the five genera of *Botryotinia, Cladosporium, Fusarium, Monilinia*, and *Hormonema*, along with unclassified genera. The raw stage shared the lowest number of OTUs with the other two stages, whereas the veraison and ripe stages shared the most OTUs. The raw stage shared a species of *Lachnum* with the veraison stage, and the species of *Penicillium* and *Perusta* with the ripe stage. The ripe stage shared 18 OTUs with the veraison stage, which were classified to the genera *Mollisia, Sporormiella, Lachnum, Phacidium, Podosphaera, Neocucurbitaria, Lapidomyces, Ophiognormonia, Aureobasidium, Melanodiplodia, Acephala*, and *Mycosymbioces*, along with unclassified genera.

### The core microbiome changed through fruit development

We identified three core OTUs across 50% of the total samples. They were classified as *Botryotinia* sp., *Cladosporium* sp., and *Monilinia gaylussaciae*. These species were the only representatives of their genus. In the raw stage, *M. gaylussaciae* and *Cladosporium* sp. were identified as core taxa. During the veraison stage, the *Botryotinia* sp. and *Cladosporium* sp. were the core OTUs, while *Cladosporium* sp. was the only core member in the ripe stage. Specific core OTUs followed a distinct dynamics along the bilberry fruit development (Fig. 1F). *M. gaylussaciae* and *Cladosporium* sp. were abundant through the developmental stages (Kruskal–Wallis, FDR-corrected *P* value of < .05), but showed a divergent trend, as *M. gaylussaciae* decreased towards the ripe stage, while the *Cladosporium* sp. became more abundant. In contrast, *Botryotinia* sp. was not abundant among the developmental stages, but persisted in the bilberry fruit throughout the bilberry fruit development. Overall, the veraison stage appeared to be a transition stage, where the core community was distinct from the other stages.

### Phenolic profile of bilberry drives the fruit-associated fungal biodiversity

**Figure 2.**
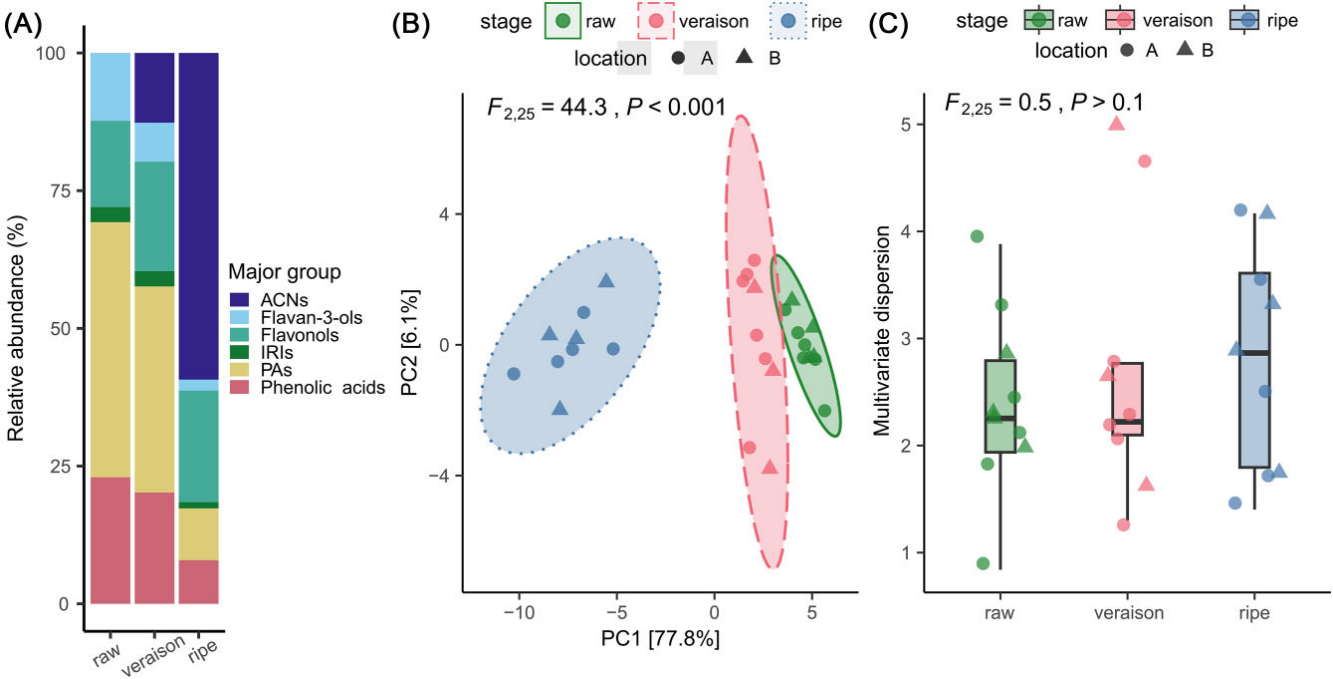
Bilberry phenolic compounds at the three developmental stages (raw, veraison, ripe). (A) Stacked bar plots of phenolic compounds classified into major groups based on their chemical structure, (B) PCA ordination, and (C) multivariate dispersion of the phenolic compounds. Ellipses denote a confidence interval of 95% (in B). Abbreviations: ACN—anthocyanin, IRI—iridoid, PA—proanthocyanidin.

The composition of soluble phenolics changed drastically from the raw to the ripe stages, with most importantly a shift from high-PA to high-ACN concentrations (Fig. 2A and Table S3). The phenolic acids (chlorogenic acid, CGA) and flavan-3-ols (epicatechin) decreased from raw to ripe stages (Fig. 2A and Table S3). The metabolic profiles of the veraison and ripe berries each demonstrated a distinct ripening stage, although they were collected simultaneously (Fig. 2A). The flavonol concentration increased from the veraison to the ripe stages mainly due to the accumulation of myricetin derivatives (Table S3).

The structure of the phenolic profile significantly differed among the fruit developmental stages (PERMANOVA global test, *F*_2,25_ = 44.3, *P* < .001, Fig. 2B), as well as in their pairwise comparisons. The phenolic profiles of the raw and ripe developmental stages distinctly differed from each other (PERMANOVA, *F*_1,17_ = 83.1, *P* < .001), followed by veraison and ripe (PERMANOVA, *F*_1,16_ = 42.7, *P* < .001), and raw and veraison stages (PERMANOVA, *F*_1,17_ = 6.7, *P* < .001). Multivariate dispersion of the bilberry phenolic profile between the developmental stages was nonsignificant (Fig. 2C).

**Figure 3.**
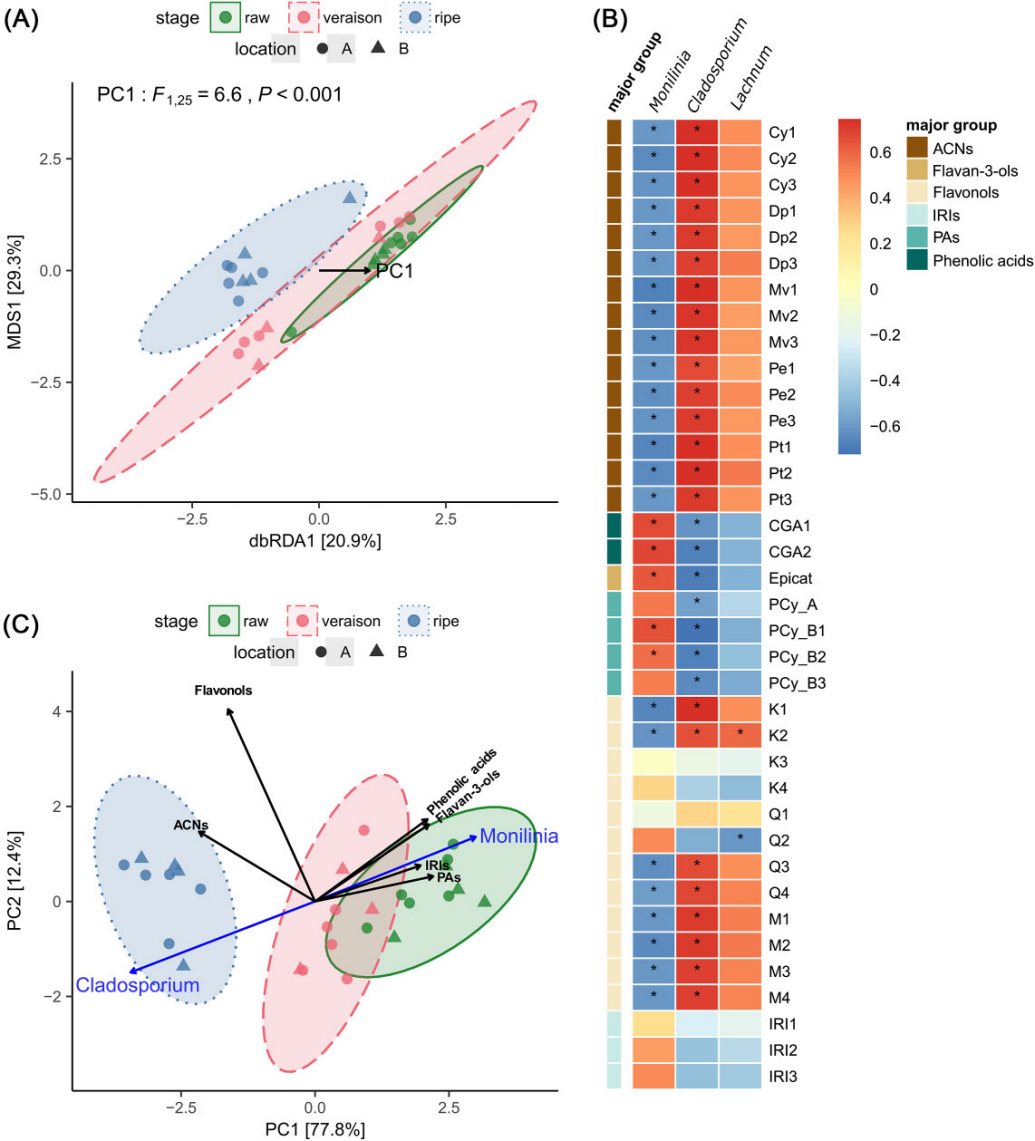
The link between phenolic compounds and fungal endophytic composition in different developmental stages (raw, veraison, ripe) of bilberries. (A) A dbRDA ordination visualizing the phenolic PC1 variable explaining the fungal community assemblages, (B) a heatmap of the Spearman correlation between the relative abundance of fungal genera and bilberry phenolic compounds, and (C) a PCA ordination of the major groups of bilberry phenolic compounds grouped by developmental stages with fitted vectors for the phenolic groups and the fungal genera. Ellipses denote a confidence interval of 95% (in A and C). Asterisks indicate significant correlation (in B). Abbreviations for phenolic compounds derivatives: Cy—cyanidin, Dp—delphinidin, Mv—malvidin, Pe—peonidin, Pt—petunidin, CGA—chlorogenic acid, Epicat—epicatechin, PA—proanthocyanidin, K—kaempferol, Q—quercetin, M—myricetin, IRI—iridoid, ACN—anthocyanin.

The dbRDA model on the fungal dRAI with the PC1 of phenolic compounds as the explanator was significant (*F*_1,25_ = 6.6, *P* < .001) and explained approximately 18% of the fungal community variation (Fig. 3A). However, the impact of the phenolic PC1 on the multivariate dispersion (or beta diversity) of the fungal community bordered significance (*P* = .05) (Table S4). The multivariate dispersion was positively driven by the fungal richness (Table S5).

The relative abundance of the genus *Monilinia* showed a strong negative correlation with several ACNs and flavonols and a positive correlation with phenolic acids, flavan-3-ols, and PAs (Fig. 3B). In contrast, the genus *Cladosporium* showed an opposite tendency. The genus *Lachnum* had a negative correlation with the flavonol compound Q2 (a quercetin derivative) and a positive correlation with the compound K2 (a kaempferol derivative). The rest of the genera had no significant correlation with the bilberry phenolic compounds. The PCA ordination for the major groups of phenolic compounds showed that ACNs and flavonols were the most abundant in the ripe bilberries concurrently with the presence of *Cladosporium* (Fig. 3C). On the other hand, phenolic acids, flavan-3-ols, PAs, and *Monilinia* were more abundant in raw bilberries. In addition, the abundance of the genus *Monilinia* was positively driven by the phenolic PC1 (Table S6), whereas it was the opposite for the genus *Cladosporium* (Table S7).

## Discussion

Endophytes are a part of the plant holobiont and can be beneficial to the host plant by promoting its ability to cope with abiotic or biotic stresses (Yuan et al. 2017, Llorens et al. 2019), by contributing to the host secondary metabolism (Pandey et al. 2016), or by improving fruit quality (Fakhro et al. 2010). However, little is known about the endophytic community in fruits over development, and the impact of fruit secondary metabolites on shaping the fruit-associated endophytic community. Therefore, we studied the fungal endophytic microbiome in the fruits of bilberry, highly rich in phenolic compounds, at three developmental stages: raw (green berries), veraison (red berries), and ripe (blue berries). Our earlier study pointed towards the availability of spores in the environment being an important factor in determining the composition of endophytic fungal communities in ripe bilberry fruits (Nguyen et al. 2024). In the current study, we propose that the change of fungal communities through the developmental stages is due to fruit chemical traits, such as the phenolic compounds. Specifically, we found that (i) the diversity of the fungal endophytes increased through the ripening stages; (ii) the fungal community composition had the greatest difference between the raw and ripe stages; (iii) each developmental stage harbored core members, and the veraison stage was a transition stage, where the core members changed; (iv) the change of bilberry phenolic profile from the raw to ripe stages shaped the community structure of the fungal endophytes and the abundance of the fungal genera *Monilinia* and *Cladosporium*, the most abundant genera in the raw and ripe bilberries, respectively.

Earlier, Tadych et al. (2012) found an increase in the fungal richness towards the ripening of cranberry ovaries, similar to our results. However, the increased species richness through development could be due to tissue age (i.e. the tissue age increases from raw to ripe stages) and depend on the sampling date. Such phenomenon has been reported in leaves (Hata et al. 1998, Osono 2008). Our data indicate that tissue age is a stronger driving factor for the increased species richness than the sampling time in bilberry fruit, as the richness differed significantly between veraison and ripe berries even though they were collected simultaneously. On the other hand, the beta diversity and the community structure differed between the raw stage and the other two stages, but not between the veraison and ripe stages, which would indicate a stronger effect of the sampling time than the tissue age.

The host phenolic compounds likely played an important role in shaping the fungal communities, because they had a statistically significant effect on the community structure, specifically, the phenolic PC1 explained 18% of the fungal community variation. Similar results have been found in leaves, where leaf traits, such as flavonoids, anthocyanins and terpenoids, were associated with the differences in foliar endophytic communities (González‐Teuber et al. 2020). In general, endophytes are suggested to go through various filters to establish in the host (Saunders et al. 2010, Van Bael et al. 2017, Zambell and White 2017): (i) abiotic factors (such as temperature and radiation), (ii) plant traits, and (iii) competitive filters (microbial species interactions). Both the selective pressure of phenolic compounds and the tolerance of fungi determine the fungal phyllosphere community composition, and the growth inhibition of each fungal species by the phenolic compounds could differ between hosts (Saunders and Kohn 2009, Saunders et al. 2010, Van Bael et al. 2017, González‐Teuber et al. 2020). For example, inhibition of fungal growth by leaf chemicals was stronger for the genera *Alternaria* and *Fusarium* than for *Cladosporium* and *Pleospora* in host trees from the temperate rainforest (González‐Teuber et al. 2020). Furthermore, a recent study on the effect of polyphenolic compounds on the growth of 15 oak-associated fungal endophytes and saprotrophic species showed that the leaf compounds had a positive effect on growth of the host-specific endophytes (Nickerson et al. 2023). Fungal detoxification of chemicals, which is based on enzymes and activation of membrane transporters, enables the efficient colonization of host tissue (VanEtten et al. 2001, Saunders and Kohn 2009), varying between fungal species (Saunders et al. 2010). In bilberry fruits, phenolic compounds accumulate in both the fruit skin and the pulp (Riihinen et al. 2008, Dare et al. 2022). Due to the antimicrobial properties (Puupponen-Pimiä et al. 2005a,b, Ermis et al. 2015) and the roles in plant defense and interaction with microbes (Mandal et al. 2010, Lattanzio 2013) of phenolic compounds, we propose that the fungal community structure of bilberry fruits changes through development due to the selection pressure on fungi by each phenolic compound, characteristic of each developmental stage.

The remaining proportion of variation in community structure not explained by the phenolic PC1 are likely other factors not examined in our study, such as tissue density and sugar content. For example, tissue density is suggested to affect the colonization of endophytes in leaves (Van Bael et al. 2017). A fungal spore on a leaf surface can germinate and then pierce the plant surface using a penetration peg (a specialized hypha) (Kankanala et al. 2007, Van Bael et al. 2017) or enter the leaf via stomata (Guimarães and Stotz 2004, Van Bael et al. 2017). Inside the leaf, fungal hyphae extension through the mesophyll is likely to be restricted by tissue density (Van Bael et al. 2017). Furthermore, root carbohydrates are suggested to shape the fungal diversity in the rhizosphere (Hadacek 2002). Accumulation of soluble sugars during fruit development (Dare et al. 2022) could also affect the fungal community in bilberry fruits.

We found the genus *Monilinia* dominant in the raw fruits, and the genus *Cladosporium* dominating in the ripe fruits. Based on the statistically significant results from the correlation test and GLMM models for these genera and phenolic profiles, we suggest that there is a link between the phenolic compounds present in each developmental stage and the fungal genera. The current literature presents the role of each phenolic compound as a defensive filter, through which each fungal strain needs to pass to establish in the host. In the case of *Monilinia*, the phenolic compounds that accumulated in the ripe fruits, specifically ACNs and flavonols, could be inhibitory. Supporting this, there is a report that pear infected with a pathogenic strain of *M. fructicola* results in the increase of flavonols in association with the plant defense (Santin et al. 2018). Another reason could be the colonization by competitors in the mature fruit, such as *Cladosporium* (Arnold et al. 2003, Pan and May 2009, Saunders et al. 2010, Adame-Álvarez et al. 2014). For *Cladosporium*, phenolic acids, flavan-3-ols, and PAs of raw fruits could act as a filter, preventing colonization. For example, essential oils of different *Nepeta* species, which contain the highest content of phenolic acids (including ferulic acid, chlorogenic acid (CGA), caffeic acid, gallic acid, and coumaric acid) compared to the other flavonoids, possess antifungal properties against the fungus *Cladosporium fluvum* (Azizian et al. 2021). On the other hand, some strains of fungi can enhance the biosynthesis of specific phenolic compounds for their own benefit. For example, the foliar endophytic fungus *Paraconiothyrium variabile* can metabolize glycosylated flavonoids of its host plant to benefit the hyphal growth of germinated spores (Tian et al. 2014). Aleynova et al. (2022) reported that the expression of phenylalanine ammonia lyase (*PAL*) was significantly induced when a non-viable product of an endophytic *Cladosporium* strain was added to a grape (*Vitis amurensis*) callus culture. PAL is the key regulating enzyme of the phenylpropanoid pathway which leads, among others, to the biosynthesis of anthocyanins, proanthocyanidins, and flavonols (Dixon 2001). Therefore, the endophytic *Cladosporium* sp. of bilberry fruits might not only possess traits to tolerate the high content of phenolic compounds in ripe fruits but also enhance the biosynthesis of these compounds.

To summarize, our findings show that the fungal endophytic communities change throughout the ripening process of bilberry fruits, where the sampling time, tissue age and fruit phenolic compounds shape the fungal communities. Especially, the genera *Monilinia* and *Cladosporium* strongly correlated with the phenolic compounds present in the raw and ripe stages, respectively. The present work is among the few studies linking host chemical traits to fungal endophytic communities, especially in fruits.

## Supplementary Material

fiae168_Supplemental_File

## Data Availability

The data of this study are openly available in the ENA at EMBL-EBI (www.ebi.ac.uk) under the accession number PRJEB61376.
