# Supplementary material for: Dynamics of fungal endophytic communities in bilberry (Vaccinium myrtillus L.) fruits through development is shaped by host phenolic compounds
Source: FEMS Microbiol Ecol. 2024 Dec 23;101(1):fiae168. doi: 10.1093/femsec/fiae168 (PMC11730187; doi:10.1093/femsec/fiae168)
Supplement: fiae168_Supplemental_File [file fiae168_supplemental_file.docx]

**Supporting information (legends)**

Fig. S1: Boxplots of the soil variables and the Wilcoxon test’s results for the soil variables between two locations.

Fig. S2: The aerial image of the sampling sites based on the map provided by the National Land Survey of Finland.

Table S1: Soil properties at two locations A and B.

Table S2: LC-MS data for phenolic compounds quantification.

Table S3: Phenolic compounds concentrations (mg/g DW) in bilberry at three developmental stages.

Table S4: Summary of the best GLMM model of the multivariate dispersion values as a function of phenolic PC1.

Table S5: Summary of the best GLMM model of the multivariate dispersion values as a function of fungal richness.

Table S6: Summary of the best GLMM model of the genus *Monilinia* abundance as a function of phenolic PC1.

Table S7: Summary of the best GLMM model of the genus *Cladosporium* abundance as a function of phenolic PC1.

Methods S1: Soil analysis

**Supplementary figures**


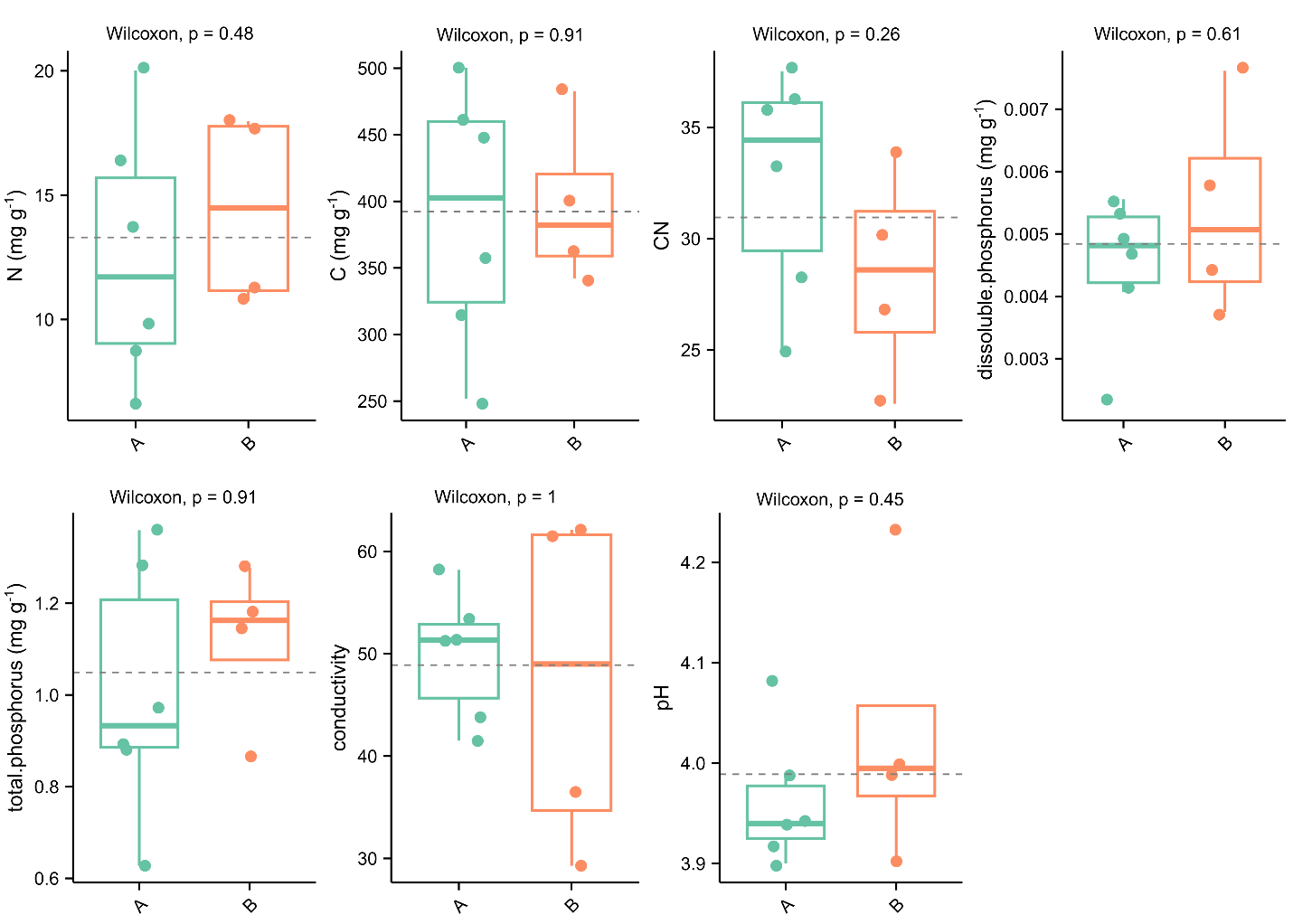


Fig S1: Boxplots of the soil variables and the Wilcoxon test’s results for the soil variables between two locations. Dash lines indicate the mean value of the variables.


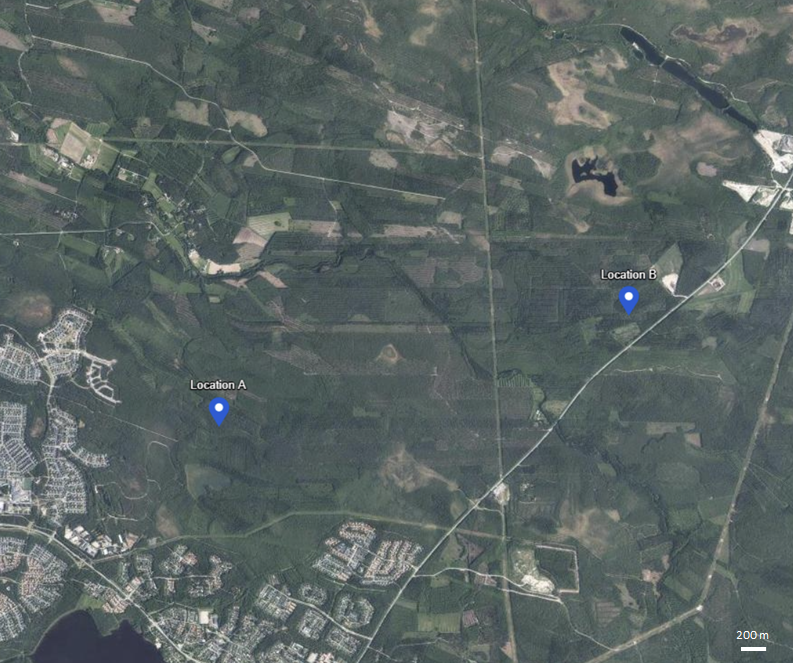


Kuivasjärvi

Fig S2: The aerial image of the sampling sites based on the map provided by the National Land Survey of Finland. Location A (65°05'12.4"N 25°29'39.3"E) and location B (65°05'46.5"N 25°34'14.9"E) are indicated.

**Supplementary tables**

Table S1: Soil properties at two locations A and B. Six and four biological replicates are analyzed for locations A and B, respectively.

| SampleID | location | N (mg g^-1^) | C (mg g^-1^) | CN | dissoluble phosphorus (mg g^-1^) | total phosphorus (mg g^-1^) | conductivity | pH |
| --- | --- | --- | --- | --- | --- | --- | --- | --- |
| A1 | A | 13.66 | 452.07 | 33.09 | 0.005 | 0.97 | 58.20 | 3.90 |
| A2 | A | 6.71 | 251.83 | 37.53 | 0.002 | 0.88 | 53.40 | 3.94 |
| A3 | A | 9.75 | 353.37 | 36.23 | 0.005 | 0.63 | 41.50 | 3.92 |
| A4 | A | 16.37 | 462.65 | 28.26 | 0.004 | 1.36 | 43.80 | 4.08 |
| A5 | A | 20.02 | 500.15 | 24.98 | 0.005 | 1.29 | 51.30 | 3.94 |
| A6 | A | 8.80 | 314.62 | 35.77 | 0.006 | 0.89 | 51.40 | 3.99 |
| B1 | B | 10.75 | 364.57 | 33.92 | 0.008 | 0.87 | 61.50 | 3.90 |
| B2 | B | 11.28 | 342.21 | 30.33 | 0.004 | 1.28 | 36.50 | 4.00 |
| B3 | B | 17.70 | 399.71 | 22.59 | 0.004 | 1.18 | 29.29 | 4.23 |
| B4 | B | 17.97 | 482.85 | 26.87 | 0.006 | 1.15 | 62.10 | 3.99 |

Table S2: LC-MS data for phenolic compounds quantification. Abbreviations: MRM- multiple reaction monitoring; RT (min) – retention time.

| Full name | Event | Ionisation Mode | Transition | RT (min) | Full name standard |
| --- | --- | --- | --- | --- | --- |
| Cyanidin 3-O- pyranoside* | MRM | + | 449→287 | 19.87; 20.99 | cyanidin 3-O- glucoside |
| Cyanidin 3-O- arabinoside | MRM | + | 419 →287 | 23.38 | cyanidin 3-O- glucoside |
| Delphinidin 3-O- pyranoside* | MRM | + | 465→303 | 15.31; 24.36 | cyanidin 3-O- glucoside |
| Delphinidin 3-O- arabinoside | MRM | + | 435→303 | 19.42, 26.37 | cyanidin 3-O- glucoside |
| Malvidin 3-O- pyranoside* | MRM | + | 493→331 | 25.75 | cyanidin 3-O- glucoside |
| Malvidin 3-O- arabinoside | MRM | + | 463→331 | 26.82 | cyanidin 3-O- glucoside |
| Peonidin 3-O- pyranoside* | MRM | + | 463→301 | 24.39; 25.32 | cyanidin 3-O- glucoside |
| Peonidin 3-O- arabinoside | MRM | + | 433→301 | 26.37 | cyanidin 3-O- glucoside |
| Petunidin 3-O- pyranoside* | MRM | + | 479→317 | 22.47 | cyanidin 3-O- glucoside |
| Petunidin 3-O- arabinoside | MRM | + | 449→317 | 24,21 | cyanidin 3-O- glucoside |
| Epicatechin | MRM | - | 289→245 | 24.25 | epicatechin |
| Procyanidin dimer B | SIM | + | 579,0 | 18.7**; 29.2 | procyanidins dimer B2 |
| Procyanidin trimer A | SIM | + | 863,0 | 19.2 | procyanidins dimer B2 |
| Procyanidin trimer B | SIM | + | 865,0 | 22.4 | Procyanidins dimer B2 |
| Kaempferol 3-O-pyranoside | MRM | -28 | 447→284 | 36.0 | hyperoside |
| Kaempferol 3-O-rutinoside | MRM | -30 | 593 -> 285 | 31.7; 33.3 | hyperoside |
| Kaempferol 3-O_glucuronide | MRM | 31 | 461 -> 285 | 36.8 | hyperoside |
| Quercetin 3-O- pyranoside | MRM | 27 | 463→301 | 31.2; 31.9; 34.0; 34.3 | hyperoside |
| Quercetin 3-O-glucuronide | MRM | 29 | 477 -> 301 | 35.2 | hyperoside |
| Myricetin 3-O-pyranoside | MRM | 26 | 479 -> 317 | 27.6; 29.2; 30.8 | hyperoside |
| Myricetin 3-O-glucuronide | MRM | 25 | 493 -> 317 | 32.0 | hyperoside |
| Caffeoylquinic acid (chlorogenic acid) | MRM | - | 353,1→191,1 | 20.7***; 25.3 | 3-O-caffeoylquinic acid |
|  |  |  |  |  |  |

*: pyranoside (galactoside and/or glucoside).

**: B2

***: 3-O-caffeoylquinic acid

Table S3: Phenolic compounds concentrations (mg/g DW) in bilberry at three developmental stages. Values are means and standard errors of approximately 10 biological replicates (6 from location A, 4 from location B). Two outliers were excluded (one ripe sample from location A, and one veraison sample from location B). The phenolic compounds were grouped into major groups based on their chemical structures.

|  |  | **mean** | | | ***SE*** | | |
| --- | --- | --- | --- | --- | --- | --- | --- |
| **Major group** | **Compounds** | **Raw** | **Veraison** | **Ripe** | ***Raw*** | ***Veraison*** | ***Ripe*** |
| Anthocyanins (ACNs) | Cyanidins | 0.00 | 2.99 | 15.89 | *0.00* | *0.33* | *0.84* |
|  | Delphinidins | 0.00 | 1.62 | 12.19 | *0.00* | *0.17* | *0.79* |
|  | Malvidins | 0.00 | 0.22 | 3.56 | *0.00* | *0.03* | *0.36* |
|  | Peonidins | 0.00 | 0.22 | 2.28 | *0.00* | *0.03* | *0.21* |
|  | Petunidins | 0.00 | 0.47 | 4.57 | *0.00* | *0.05* | *0.34* |
| Flavonols | Kaempferols | 0.15 | 0.20 | 0.30 | *0.02* | *0.03* | *0.02* |
|  | Quercetins | 6.50 | 6.44 | 6.54 | *0.36* | *0.68* | *0.47* |
|  | Myricetins | 0.08 | 0.70 | 4.33 | *0.01* | *0.05* | *0.27* |
| Proanthocyanidins (PAs) | Proanthocyanidins A | 18.16 | 11.85 | 4.29 | *0.88* | *0.73* | *0.34* |
|  | Proanthocyanidins B | 9.94 | 7.72 | 3.02 | *1.13* | *0.86* | *0.38* |
| Phenolic acids | Chlorogenic acids | 11.62 | 8.81 | 5.14 | *0.52* | *0.66* | *0.25* |
| Flavan-3-ols | Epicatechin | 5.28 | 2.61 | 1.09 | *0.30* | *0.21* | *0.08* |
| Iridoids (IRIs) | Iridoids | 1.67 | 1.46 | 0.89 | *0.12* | *0.06* | *0.07* |
| **Major group** |  |  |  |  |  |  |  |
| Anthocyanins (ACNs) |  | 6.73 | 12.87 | 49.65 | *0.37* | *1.11* | *2.76* |
| Flavonols |  | 6.72 | 7.35 | 11.16 | *0.37* | *0.72* | *0.64* |
| Proanthocyanidins (PAs) |  | 28.11 | 19.57 | 7.31 | *1.42* | *1.49* | *0.38* |
| Phenolic acids |  | 11.62 | 8.81 | 5.14 | *0.52* | *0.66* | *0.25* |
| Flavan-3-ols |  | 5.28 | 2.61 | 1.09 | *0.30* | *0.21* | *0.08* |
| Iridoids (IRIs) |  | 1.67 | 1.46 | 0.89 | *0.12* | *0.06* | *0.07* |
| TOTAL |  | 6.73 | 12.87 | 49.65 | *0.37* | *1.11* | *2.76* |

Table S4: Summary the best GLMM model of the multivariate dispersion values as a function of phenolic PC1. Predictor variables included in the model are denoted as fixed effects.

| m <- glmmTMB(multivariate dispersion ~ PC1 + offset(log sequencing depth) + (1\|location), data = metadata , family =gaussian) | | | | | |
| --- | --- | --- | --- | --- | --- |
|  | AIC | BIC | logLik | deviance | df.resid |
|  | 126.6 | 131.9 | -59.3 | 118.6 | 26 |
| Random effects | Groups | Name | Variance | Std.Dev. |  |
|  | location | (Intercept) | 0.14 | 0.38 |  |
|  | Residual |  | 3.76 | 1.94 |  |
|  | Number of obs: 28, groups: location, 2 | | | | |
|  |  |  |  |  |  |
| Fixed effects |  | Estimate | Std. Error | z value | Pr(>\|z\|) |
|  | (Intercept) | -6.39 | 0.46 | -13.76 | <0.001 |
|  | PC1 | -0.13 | 0.07 | -1.93 | 0.05 |

Table S5: Summary the best GLMM model of the multivariate dispersion values as a function of fungal richness. Predictor variables included in the model are denoted as fixed effects.

| m <- glmmTMB(multivariate dispersion ~ richness + offset(log sequencing depth) + (1\|location), data = metadata , family =gaussian) | | | | | |
| --- | --- | --- | --- | --- | --- |
|  | AIC | BIC | logLik | deviance | df.resid |
|  | 114.1 | 119.5 | -53.1 | 106.1 | 26 |
| Random effects | Groups | Name | Variance | Std.Dev. |  |
|  | location | (Intercept) | 3.31e-09 | 5.75e-05 |  |
|  | Residual |  | 2.36 | 1.54 |  |
|  | Number of obs: 28, groups: location, 2 | | | | |
|  |  |  |  |  |  |
| Fixed effects |  | Estimate | Std. Error | z value | Pr(>\|z\|) |
|  | (Intercept) | -8.69 | 0.58 | -15.09 | <0.001 |
|  | richness | 0.26 | 0.06 | 4.69 | <0.001 |

Table S6: Summary the best GLMM model of the genus Monilinia abundance as a function of phenolic PC1. Predictor variables included in the model are denoted as fixed effects. The zero-inflated model was used because it passed the model validation while the non-zero-inflated model did not.

| *Monilinia* | | | | | |
| --- | --- | --- | --- | --- | --- |
| m <- glmmTMB (abundance ~ PC1 + offset(log sequencing depth) + (1\|location), zi = ~PC1, data = metadata , family =nbinom2) | | | | | |
|  | AIC | BIC | logLik | deviance | df.resid |
|  | 390 | 398 | -189 | 378 | 22 |
| Random effects | Groups | Name | Variance | Std.Dev. |  |
|  | location | (Intercept) | 1.25e-09 | 3.54e-05 |  |
|  |  |  |  |  |  |
|  | Number of obs: 28, groups: location, 2 | | | | |
|  |  |  |  |  |  |
| Fixed effects | | Estimate | Std. Error | z value | Pr(>\|z\|) |
|  | (Intercept) | -1.74 | 0.22 | -8.02 | <0.001 |
|  | PC1 | 0.26 | 0.05 | 5.60 | <0.001 |
|  |  |  |  |  |  |
| Zero-inflat model | | Estimate | Std. Error | z value | Pr(>\|z\|) |
|  | (Intercept) | -0.31 | 0.42 | -0.73 | >0.1 |
|  | PC1 | -0.17 | 0.08 | -2.10 | <0.05 |

Table S7: Summary the best GLMM model of the genus Cladosporium abundance as a function of phenolic PC1. Predictor variables included in the model are denoted as fixed effects.

| *Cladosporium* | | | | | |
| --- | --- | --- | --- | --- | --- |
| m <- glmmTMB(abundance ~ PC1 + offset(log sequencing depth) + (1\|location), data = metadata , family =nbinom2) | | | | | |
|  | AIC | BIC | logLik | deviance | df.resid |
|  | 417.4 | 422.7 | -204.7 | 409.4 | 24 |
| Random effects | Groups | Name | Variance | Std.Dev. |  |
|  | location | (Intercept) | 0.94 | 0.97 |  |
|  |  |  |  |  |  |
|  | Number of obs: 28, groups: location, 2 | | | | |
|  |  |  |  |  |  |
| Fixed effects | | Estimate | Std. Error | z value | Pr(>\|z\|) |
|  | (Intercept) | -4.00 | 0.80 | -5.03 | <0.001 |
|  | PC1 | -0.24 | 0.09 | -2.72 | <0.01 |

**Method S1: Soil analysis**

Soil samples were collected with the sampling of green berries (on 5 July 2021). We used a soil drill to dig the soil up to a depth of 15 cm, then the humus layer was collected into a zip bag. Four soil cores were homogenized for each replicate. The soil samples were stored on ice (+4^o^C) for transportation and later in -20^o^C at the laboratory for further analysis.

We analyzed conductivity, pH, and the contents (mg g^-1^) of carbon (C), nitrogen (N), and total phosphorus following the protocol by (Nguyen *et al.*, 2024). To measure the dissoluble phosphorus, 20 ml of soil was weighed and shaken in 100 ml ammonium acetate for 1 h. The mixture was allowed to rest for at least 30 min, then the supernatant was filtered through the fiberglass paper (Whatman 42). Five ml of the filtered solution was mixed with 5 ml of a combined reagent (1.5% (w/v) ascorbic acid, 2% (w/v) ammonium molybdate, 4.5 M H_2_SO_4_, and 0.05% (w/v) antimony potassium tartrate), then milli-Q water was added to the solution to the final volume of 50 ml. The final solution was used for phosphorus analysis with a UV-visible spectrophotometer (Shimadzu UV-1700). The concentration of phosphorus (mg g^-1^) was calculated based on the formula: $C=\frac{C^{'}.V.df}{W}$ with *C* – concentration (mg g^-1^), *C’ –* concentration given by the spectrophotometer (mg l^-1^), *V* *–* total volume (l), *df* *–* dilution factor, *W* *–* weight of starting material (g).

References

**Nguyen MP, Lehosmaa K, Toth K, Koskimäki JJ, Häggman H, Pirttilä AM**. **2024**. Weather in two climatic regions shapes the diversity and drives the structure of fungal endophytic community of bilberry (Vaccinium myrtillus L.) fruit. *Environmental Microbiome* **19**: 1–12.
